# Psychological and relational adjustment under stress: the mediating role of emotion regulation in parents' functioning during the COVID-19 crisis

**DOI:** 10.3389/fpsyg.2025.1678034

**Published:** 2025-10-07

**Authors:** Alessandro Taurino, Rosalinda Cassibba, Cristina Semeraro, Gabrielle Coppola, Maria Dentamaro, Pasquale Musso

**Affiliations:** ^1^Department of Education, Psychology, Communication, University of Bari Aldo Moro, Bari, Italy; ^2^School Istituto Comprensivo di Stato “Mazzini-Modugno”, Bari, Italy

**Keywords:** COVID-19, emotion regulation, parents, psychological wellbeing, relational functioning

## Abstract

**Introduction:**

Periods of intense, prolonged stress, such as the COVID-19 pandemic, can undermine parents' psychological and relational adjustment. Guided by the Family Stress Model and transactional theory, we examined whether specific cognitive emotion-regulation strategies account for the association between pandemic stressors and parental functioning.

**Methods:**

Between April and May 2021, 212 parents of school-aged children in Southern Italy (89.6% mothers; *M*_*age*_ = 42.6 years) completed an online survey that assessed perceived viral threat, pandemic-related financial hardship, COVID-19 psychological impact, five cognitive emotion-regulation strategies (positive reappraisal, putting into perspective, planning, rumination, catastrophizing), psychological wellbeing (positive affect, flourishing), and relational functioning (parent-child closeness, parent-teacher joining).

**Results:**

Structural equation modeling with robust maximum likelihood estimation controlled for parent age, gender, direct COVID-19 exposure, and socioeconomic status. The final model demonstrated excellent fit. Perceived threat and psychological impact predicted poorer wellbeing indirectly through higher catastrophizing and, only for psychological impact, lower planning. Catastrophizing and planning fully mediated these pathways, whereas rumination and other adaptive strategies were non-significant. Financial hardship was unrelated to emotion-regulation strategies yet directly associated with poorer relational functioning.

**Discussion:**

These findings highlight catastrophizing as a maladaptive and planning as an adaptive pathway through which pandemic stress translates into parental adjustment difficulties, informing the design of targeted coping-skills programs and economic relief policies.

## 1 Introduction

Periods marked by intense and prolonged stress, such as health emergencies or large-scale societal disruptions, pose serious challenges to individuals' psychological wellbeing and relational functioning by placing considerable demands on their adaptive resources (e.g., Musso et al., 2024). The COVID-19 pandemic has emerged as one of the most recent and unprecedented global stressors, characterized by these conditions and far- reaching implications (Brooks et al., 2020; Catling, 2023; Horesh and Brown, 2020; World Health Organization, 2022). Although its immediate health threat has largely receded, the benefit of temporal distance allows the pandemic to be regarded as a unique “stress laboratory”, that has offered valuable insights into the psychological mechanisms underlying widespread adaptation to highly stressful circumstances. In particular, it has deepened our understanding of how individuals and families respond to acute stress, shedding light on the processes that foster resilience across multiple levels of functioning. Current literature supports this perspective. For example, (Prime et al. 2020) emphasized that pandemic-related disruptions (such as social isolation, financial strain, and increased caregiving demands) produced cascading effects, that initially undermined caregivers' wellbeing and subsequently affected family processes and children's adjustment. More recently, scholars have framed the pandemic as a prolonged stress context that reveals heterogeneity in resilience trajectories and highlights the role of regulatory flexibility in coping with complex adversity (Bonanno et al., 2024; Landi et al., 2022; Zarowsky and Rashid, 2023).

International research has consistently documented substantial psychological distress associated with the pandemic, including increased anxiety, depressive symptoms, and emotional dysregulation (Al-Akashee et al., 2024; Xiong et al., 2020). In this context, parents have emerged among the groups most vulnerable to pandemic-related strain, experiencing significant disruptions in their psychological wellbeing and in the quality of their relationships, both within the family and across broader social networks (Cassinat et al., 2021; Marchetti et al., 2020; Spinelli et al., 2020). As a matter of fact, parents' wellbeing and day-to-day functioning appeared especially sensitive to the pressures that accompanied the pandemic: heavier caregiving duties, economic insecurity, dwindling social support, and persistent worries about personal and family safety (Prime et al., 2020; Restubog et al., 2020).

These burdens, however, did not weigh on every society in the same way. Contextual forces shaped families' experiences, and Italy, first and hardest hit among European countries, provides a particularly vivid case of how pandemic-related stressors were lived. The country imposed stringent public health measures, including prolonged lockdowns and strict social distancing mandates, which had profound psychological and relational consequences for families (Morelli et al., 2020; Spinelli et al., 2020). Studies report that Italian parents perceived particularly high levels of stress, driven by extended home confinement, increased domestic duties, difficulties managing remote schooling, and heightened concerns for family health (e.g., Cusinato et al., 2020; Marchetti et al., 2020). These stressors also extended beyond the household, affecting parents' relationships with teachers, whose roles were abruptly transformed due to the transition to distance learning (Cassinat et al., 2021). Such disruptions frequently undermined the quality of collaboration between families and schools, with potential consequences for children's developmental outcomes (Mangiavacchi et al., 2021; Prime et al., 2020).

In light of these documented impacts, recent literature has underscored the importance of identifying the psychological mechanisms that explain why and how pandemic-related stressors have affected parents' psychological and relational functioning. A central pathway involves the way individuals interpret and manage adverse experiences, hence the focus on emotion regulation. According to Gross's (2014) process-oriented model, emotion regulation encompasses cognitive change, including both adaptive strategies—such as positive reappraisal and plan-focused refocusing—and maladaptive strategies, including rumination and catastrophizing. A substantial body of evidence indicates that adaptive strategies are linked to better psychological wellbeing, whereas maladaptive strategies are associated with heightened distress (e.g., Balzarotti et al., 2016; Garnefski et al., 2005; Garnefski and Kraaij, 2018; Ramos-Galarza et al., 2024). In particular, the cognitive emotion regulation framework developed by (Garnefski et al. 2001) emphasizes the interpretive processes through which individuals cognitively frame stressful events, shaping emotional reactions and behavioral responses. For example, (Manesh and Malak 2025) showed that emotion regulation mediates the impact of early neglect on risky behaviors among a sample of prisoners. Thus, emotion regulation may serve as a crucial mechanism explaining whether pandemic-related stressors resulted in psychological impairment or, alternatively, fostered resilient functioning among parents (Restubog et al., 2020).

Empirical work conducted during the COVID-19 outbreak, both cross-sectional and longitudinal, has corroborated this mechanism, underscoring the central mediating role of emotion regulation in high-stress conditions. Specifically, studies indicate that persons, including parents, who relied on maladaptive strategies reported steeper increases in anxiety and depressive symptoms throughout the health emergency, whereas adaptive strategies were linked to reduced psychological distress and more positive adjustment (Prikhidko et al., 2020; Wang et al., 2021; Xu et al., 2020; Zacher and Rudolph, 2021). These findings suggest that examining the mediational function of emotion regulation may offer critical insight into why some parents maintained adequate functioning despite the intense stressors of the pandemic, while others experienced significant psychological difficulties.

However, the importance of emotion regulation extends beyond individual psychological outcomes to encompass key relational dimensions: effective emotion regulation plays a crucial role in sustaining supportive and nurturing parent-child relationships during times of stress, fostering emotional warmth, responsiveness, and sensitivity, whereas parents who rely on maladaptive strategies are more likely to experience stressed relational dynamics, often marked by conflict, misunderstanding, and emotional detachment (e.g., Cole and Hollenstein, 2018; Cohen et al., 2022; Paley and Hajal, 2022; Rutherford et al., 2015; Zimmer-Gembeck et al., 2022). Moreover, given the pivotal role schools played during the pandemic in remotely supporting families, the quality of parent-teacher relationships might be also shaped by parents' emotional coping strategies (Adams and Christenson, 2000; Carrión Martínez et al., 2021; Gershy and Katz, 2023; Keengwe and Onchwari, 2024). Parents who employed adaptive regulation strategies tended to show greater collaboration and more effective communication with teachers, thereby promoting both educational and psychological benefits for their children (Kim and Asbury, 2020).

In summary, the existing literature suggests that cognitive emotion regulation strategies may function as key mediating mechanisms through which pandemic-related stressors impacted parents' psychological wellbeing and relational functioning. However, despite significant progress in understanding these links, important gaps remain. Notably, few studies have simultaneously investigated multiple pandemic-specific stressors—such as perceived threat, financial/material hardship, and psychological impact—in relation to both psychological and relational adjustment, within a unified emotion regulation framework. Furthermore, the complex interplay between individual psychological functioning and relational contexts, including parent-child and parent-teacher relationships, requires further exploration to inform the development of integrated theoretical models supported by robust empirical evidence.

To address these gaps, recent scholarship has highlighted the need for integrative models that simultaneously consider psychological and interpersonal dimensions to capture the full complexity of adaptation processes under stress (Liu et al., 2017; Prime et al., 2020). Comprehensive theoretical frameworks, such as the Family Stress Model (Masarik and Conger, 2017), posit that external stressors (e.g., economic hardship, the spread of COVID-19), together with their immediate subjective appraisals (ranging from directly related perceived threat to more diffuse personal distress), affect family functioning primarily through the mediation of organized intrapsychic mechanisms, including emotion regulation. According to this model, parents' emotional and cognitive responses to stress might be critical in determining their capacity to sustain psychological wellbeing, manage caregiving tasks effectively, and maintain positive interactions both within the family and with their children's educational contexts. Applying such integrative frameworks to the context of the COVID-19 pandemic may help clarify how multiple concurrent stressors shaped both individual and relational outcomes through specific emotion regulation pathways.

### 1.1 The present study

Building on this background, the present study examined how multiple stressors experienced during the COVID-19 crisis were associated with parents' psychological and relational adjustment, with a particular emphasis on the mediating role of emotion regulation strategies. In line with the theoretical frameworks described above, pandemic-related stressors were conceptualized as risk factors potentially relevant to parental functioning. Specifically, we focused on three central dimensions of stress that were widely reported during the pandemic and found to be especially salient for families (Conway et al., 2020): (1) perceived threat related to COVID-19, (2) financial and material hardship stemming from the pandemic, and (3) the negative psychological impact directly associated with the health crisis. Perceived threat refers to the subjective experience of fear and vulnerability associated with the presence of the virus and its potential consequences (Conway et al., 2020); financial and material difficulties encompass economic strain, income loss, and limited access to essential goods, conditions that have been shown to significantly affect parental wellbeing and compromise family stability (Gassman-Pines et al., 2020; Prime et al., 2020); and psychological impact captures lived sense of pandemic-linked emotional stress, manifested in elevated depressive mood and broad psychological discomfort, directly attributable to COVID-19 conditions (Cusinato et al., 2020).

Parents' adjustment was analyzed across two theoretically grounded domains. First, consistent with contemporary accounts of wellbeing, we adopted a dual-facet framework for psychological wellbeing. Specifically, we used hedonic wellbeing to denote subjective wellbeing—affective balance and felt happiness—whereas eudaimonic wellbeing referred to purpose, growth, autonomy, and optimal psychological functioning (Diener, 2009; Ryff et al., 2021). This distinction is pertinent in family-stress contexts, where stressors may co-occur with shifts in momentary affect (hedonic) and in broader processes of meaning and functioning (eudaimonic). Throughout the manuscript, psychological wellbeing indicates a latent construct comprising one hedonic indicator (positive affect) and one eudaimonic indicator (flourishing), as detailed in the Measures section. Second, we defined relational functioning as the perceived quality of parents' relationships with their children and with teachers, two critical social contexts markedly affected by the pandemic (Mangiavacchi et al., 2021; Spinelli et al., 2020).

Critically, this study investigated whether cognitive emotion regulation strategies, as conceptualized by Garnefski and Kraaij (2006), statistically mediated the associations between the above stressors and parental adjustment. As noted earlier, the cognitive emotion regulation framework distinguishes between adaptive and maladaptive strategies that individuals may employ when confronted with negative or threatening experiences. These strategies have been widely associated with both psychological symptoms and relational quality in contexts of elevated stress (Aldao et al., 2010; Garnefski et al., 2002). However, their specific mediating role in the context of pandemic-related parental functioning remains underexplored, as already outlined above.

**Figure 1:**
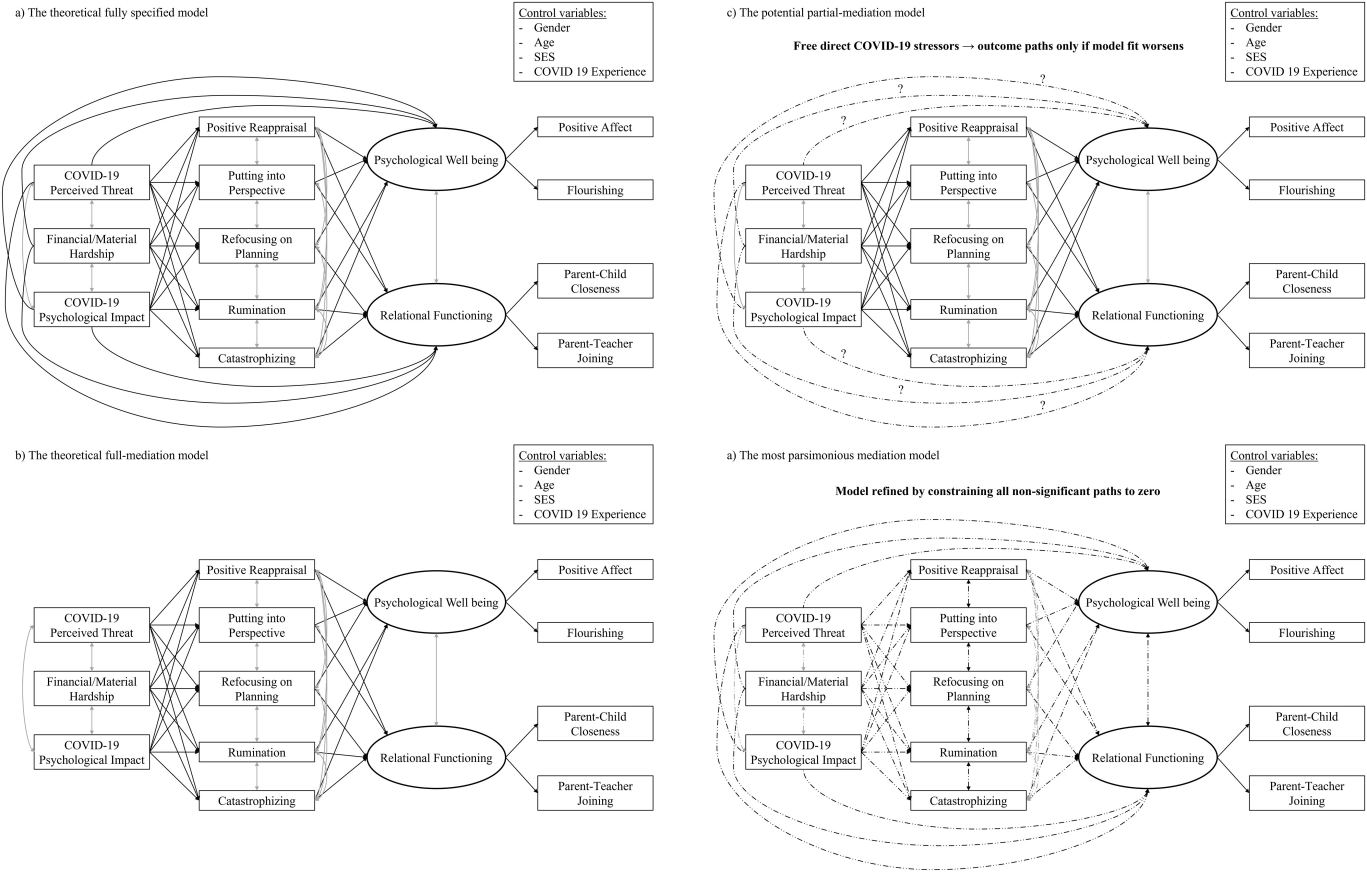
Mediation models. **(a)** The theoretical fully specified model. **(b)** The theoretical full-mediation model. **(c)** The potential partial-mediation model. **(d)** The most parsimonious modulation model.

Accordingly, we tested a theoretical model (see Figure 1) in which the three pandemic-related stressors were modeled as having direct and indirect associations with parental psychological and relational adjustment via five cognitive emotion regulation strategies, three adaptive (positive reappraisal, putting into perspective, refocusing on planning) and two maladaptive (rumination and catastrophizing). By identifying these specific regulatory processes through which pandemic-related stress influenced parental functioning, this study aimed to offer both theoretical insight and practical implications for the design of prevention and intervention strategies that promote resilience in the face of acute and future stressors. Based on their potential relevance as background factors, the model also controlled for parent age, gender (mother vs. father), direct COVID-19 experience, and socioeconomic status.

## 2 Method

### 2.1 Participants

A total of 212 parents of school-aged children participated in the study. Participants were required to be primary caregivers: most respondents were mothers (89.6%), with a mean age of 42.58 years (*SD* = 5.53); fathers reported a mean age of 48.59 years (*SD* = 6.30). All participants had at least one child attending a public school in Southern Italy (Apulia region), across two educational institutions encompassing preschool, primary, and lower secondary levels. The majority of responses were collected from a larger urban area (85.4%), with the remainder from a smaller suburban municipality (14.6%). These two schools were selected to capture a broader territorial and sociodemographic variation within the Apulian context. The higher number of participants from the urban-based institution reflects both the larger student population and the wider range of school levels represented there.

Respondents had an average of 1.37 children enrolled in the school where data were collected (*SD* = 0.53). A closer look reveals that 65.1% of parents had one child in the sample, 32.5% had two, and only 2.4% reported three. As for school level, 44.8% of the children attended primary classes, 20.8% were in lower-secondary (middle) school, and 10.8% were enrolled in preschool. Additionally, 16% of parents reported having children in both primary and middle school, while 7.6% had children in both preschool and primary school.

Parents' self-reported socioeconomic status (SES) was assessed using a 5-point subjective scale ranging from 1 (*Poor*) to 5 (*Excellent*). The mean SES score was 2.95 (*SD* = 0.79), with the majority of participants placing themselves in the midrange categories (46.7% selected *Fair*, 28.8% *Good*, and 21.7% *Very good*). Subjective SES has been widely used in literature as a valid indicator of perceived economic and social standing and has been shown to predict a range of psychological and health outcomes beyond objective measures such as income or education (e.g., Adler et al., 2000; Cundiff and Matthews, 2017). With regard to COVID-19 exposure, only a minority of the sample (10.4%) reported direct personal experience with the virus, while the majority (89.6%) did not.

### 2.2 Procedure

Data were collected in April and May 2021, a period characterized by partial school closures, hybrid learning arrangements, and persistent COVID-related stressors in Italy. Participants completed an anonymous online questionnaire designed to assess key domains of parental functioning, including perceived COVID-19-related stressors, emotion regulation strategies, psychological wellbeing, and the quality of relationships with children and teachers. The survey was administered through internal school communication channels and virtual platforms. Participation was voluntary, and informed consent was obtained from all respondents prior to data collection. No personally identifying information was collected. The study adhered to the ethical principles of the Declaration of Helsinki and received approval from the institutional ethics committee (protocol code ET-20-06).

### 2.3 Measures

To assess individual and relational functioning during the COVID-19 pandemic, participants completed validated self-report instruments adapted, when necessary, to the pandemic context. Participants responded to each scale with reference to their current experience.

#### 2.3.1 Perceived pandemic-related stressors

To assess parents' pandemic-related stressors, we administered items adapted from Conway et al. (2020 for Italian applications, see Baldner et al., 2022; Paleari et al., 2023), who developed a comprehensive set of indicators to evaluate cognitive and emotional responses to COVID-19. Guided by this framework, we measured three domains: (a) perceived threat of the virus (3 items; e.g., “I am afraid of the coronavirus [COVID-19]”), (b) perceived financial and material consequences (4 items; e.g., “The coronavirus [COVID-19] has impacted me negatively from a financial point of view”), and (c) perceived psychological impact (2 items; e.g., “The coronavirus [COVID-19] outbreak has impacted my psychological health negatively”). Items were rated on a 5-point Likert scale from 1 (*Not at all true for me*) to 5 (*Absolutely true for me*). These domains capture key dimensions of pandemic-related stress and were selected for their documented relevance to psychological functioning during health emergencies (e.g., Varese et al., 2025). Internal consistency in the present sample ranged from acceptable to excellent, with Cronbach's α = 0.88 for perceived threat, 0.85 for financial and material consequences, and 0.86 for psychological impact, supporting the reliability of the three subscales. For each stressor scale, we averaged the item scores to obtain a total, with higher totals reflecting higher levels on the measured dimension.

#### 2.3.2 Cognitive emotion regulation

To assess parents' use of cognitive emotion regulation strategies in response to pandemic events, we used the short version of the Cognitive Emotion Regulation Questionnaire (CERQ-short; Garnefski and Kraaij, 2006; for the Italian validation, see Cerolini et al., 2022), evaluating the typical cognitive mechanisms individuals employ to manage negative emotions, particularly following highly threatening or uncontrollable experiences. For this study, five subscales, each composed of two items rated on a 5-point Likert scale from 1 (*Almost never*) to 5 (*Almost always*), were selected to represent both adaptive and maladaptive strategies, based on their established theoretical relevance and empirical associations with adjustment outcomes (Garnefski et al., 2001; Aldao et al., 2010). On the adaptive side, positive reappraisal (e.g., “I think I can learn something from the situation”) reflects the tendency to reinterpret stressful events as opportunities for personal growth and has been consistently linked to reduced anxiety and enhanced wellbeing, including during the COVID-19 pandemic (see, for example, Rodas et al., 2022). Putting into perspective (e.g., “I tell myself that there are worse things in life”) captures the ability to down-regulate emotional reactivity by comparing the current situation with more severe experiences, whereas refocusing on planning (e.g., “I think about a plan of what I can do best”) denotes an active coping effort to devise concrete action plans and foster a sense of control. On the maladaptive side, rumination (e.g., “I am preoccupied with what I think and feel about what I have experienced”) involves a perseverative focus on one's emotional state, a pattern linked to heightened vulnerability to anxiety, depression, and impaired emotion regulation (Nolen-Hoeksema et al., 2008). Finally, catastrophizing (e.g., “I continually think how horrible the situation has been”) reflects the tendency to amplify the perceived severity of the stressor and anticipate worst-case outcomes, which has been associated with greater emotional distress and reduced resilience (Garnefski et al., 2002; Martin and Dahlen, 2005). Internal consistency for the selected subscales in the present sample ranged from acceptable to excellent, with Cronbach's α = 0.90 for positive reappraisal, 0.85 for putting into perspective, 0.65 for refocusing on planning, 0.79 for rumination, and 0.87 for catastrophizing. For each subscale, we averaged the item scores to obtain a total, with higher totals reflecting higher levels on the measured dimension.

#### 2.3.3 Psychological wellbeing

To assess parents' psychological wellbeing, we adopted a multidimensional approach encompassing both hedonic and eudaimonic aspects of functioning (Ryff et al., 2021), as defined above. The hedonic component was measured using a subset of five positively valenced items from the Short-Form of the Positive and Negative Affect Schedule (SF-PANAS; Thompson, 2007; Watson et al., 1988; for the Italian adaptation, see Cattelino et al., 2023; Terraciano et al., 2003). Participants rated how frequently they had experienced each emotion (e.g., “enthusiastic”, “inspired”) on a 5-point Likert scale, ranging from 1 (*Never*) to 5 (*Always*). This very brief PANAS version, focusing exclusively on positive affect, demonstrated excellent internal consistency in the current sample (Cronbach's α = 0.87). The eudaimonic component was measured through the 8-item Flourishing Scale (FS; Diener et al., 2010; for the Italian adaptation, see Di Fabio, 2016; Giuntoli et al., 2017). This scale taps perceived competence, meaningful engagement, self-acceptance, optimism, and supportive relationships (e.g., “I lead a purposeful and meaningful life,” “My social relationships are supportive and rewarding”). Participants indicated their level of agreement on a 7-point scale ranging from 1 (*Strongly disagree*) to 7 (*Strongly agree*). The FS demonstrated excellent internal reliability in the current sample (Cronbach's α = 0.93), supporting its validity as an indicator of eudaimonic wellbeing. For both scales, we averaged the item scores to obtain a total, with higher totals reflecting higher levels on the measured dimension.

#### 2.3.4 Relational functioning

To evaluate the relational functioning, we assessed two primary domains, parent-child relationship and the parent-teacher relationship, both through parent-reported measures. The quality of the parent-child relationship was evaluated using the closeness items from the Child-Parent Relationship Scale—Short Form (CPRS-SF; Pianta, 1992; Driscoll and Pianta, 2011; see Lionetti et al., 2023, for Italian use), which capture parents' perceptions of warmth, affection, and open communication within the relationship (7 items, e.g., “I share an affectionate, warm relationship with my child”, “If upset, my child seeks comfort from me”). Each item was rated by parents on a 5-point Likert scale, ranging from 1 (*Definitely does not apply*) to 5 (*Definitely applies*). The quality of the parent-teacher relationship was assessed using items from the Joining subscale of the Parent-Teacher Relationship Scale (PTRS; Vickers and Minke, 1995), which measures parents' perceptions of emotional closeness, mutual trust, and shared goals in the relationship with their child's teacher (e.g., “We trust each other”, “We have common expectations for the child”). This dimension has been shown to reflect the affective and collaborative aspects of family-school partnerships and has been adopted in previous studies to evaluate relational functioning in educational settings (for application in the Italian context, see Bosoni, 2024). Parents responded to each item using a 5-point Likert scale ranging from 1 (*Almost never*) to 5 (*Almost always*). In the present sample, both relational scales showed excellent internal consistency with Cronbach's α = 0.88 and 0.89 respectively, supporting their reliability as indicators of child-parent and parent-teacher relational quality. For both scales, we averaged the item scores to obtain a total, with higher totals reflecting higher levels on the measured dimension.

### 2.4 Data analytic plan

We performed preliminary analyses using *SPSS Version 29.0* to inspect descriptive statistics and bivariate correlations among all study variables. Means, standard deviations, and Pearson correlation coefficients were computed to assess the direction and magnitude of associations between COVID-19 stressors, cognitive emotion-regulations strategies, and personal wellbeing and relational functioning. We also screened univariate and multivariate outliers and assumptions of normality.

To test the conceptual mediation model, we estimated a structural equation model in *Mplus Version 7* (Muthén and Muthén, 2012) that included: (a) the three perceived stressors related to the COVID-19 pandemic, specifically perceived threat, financial/material strain, and psychological impact; (b) the two domains of adjustment, namely psychological wellbeing and relational quality, both modeled as latent factors with hedonic and eudaimonic indicators for the former and parent-child and parent-teacher closeness for the latter; and (c) the five dimensions of cognitive emotion regulation (positive reappraisal, putting into perspective, refocusing on planning, rumination, and catastrophizing), entered as simultaneous mediators to explain the links between perceived stress and adjustment outcomes. Covariates were parent age, gender (mother vs. father), direct COVID-19 experience, and socioeconomic status. Analyses were conducted with the robust maximum likelihood estimator (MLR), which corrects standard errors and fit indices for non-normality. The significance of indirect effects was evaluated using model-based estimates and their associated *p*-values.

The analytic strategy involved four sequential steps (see Figure 1). First, we estimated a model in which all paths—from perceived stressors to emotion regulation strategies to psychological and relational adjustment—were freely estimated, including both direct and indirect effects. Second, we tested a full mediation model, a more constrained specification in which the direct paths between perceived stressors and psychological and relational adjustment were set to zero, so that effects could operate only via emotion regulation strategies. Third, we estimated a partial mediation model by freeing one or more direct effects if the full-mediation model showed a significant worsening of fit. Fourth, we refined the model by constraining all non-significant paths to zero, resulting in a final parsimonious solution. Standardized coefficients (β) are reported for all direct and indirect paths. Model fit was evaluated with the chi-square statistic (χ^2^), Comparative Fit Index (CFI), Root Mean Square Error of Approximation (RMSEA), and Standardized Root Mean Square Residual (SRMR). Fit thresholds followed conventional cutoffs (e.g., Kline, 2015): a non-significant χ^2^ with *p* > 0.05 (bearing in mind that this test can reject the null with large samples or complex models), CFI ≥ 0.95, RMSEA ≤ 0.06, and SRMR ≤ 0.08. Nested models were compared using the scaled χ^2^ difference test (Satorra and Bentler, 2001), supplemented by changes (Δ) in CFI, RMSEA, and SRMR. Consistent with Chen's (2007) recommendations (see also Kline, 2015), a more restrictive model was considered to exhibit significantly worse fit only when at least two of the following four criteria were met: (a) a significant scaled Δχ^2^ (*p* < 0.05); (b) ΔCFI ≤ – 0.010; (c) ΔRMSEA ≥ 0.015; and (d) ΔSRMR ≥ 0.010.

## 3 Results

### 3.1 Preliminary analyses and descriptive statistics

Table 1 presents means, standard deviations, skewness, and kurtosis for all study variables, and Table 2 shows the zero-order Pearson correlations. Because the online questionnaire required a response to virtually every item, no missing data were present. Among the three COVID-19 stressors, COVID-19 Perceived Threat had the highest mean, *M* = 3.29, *SD* = 0.97, followed by Psychological Impact (*M* = 1.98, *SD* = 0.98) and Financial/Material Hardship (*M* = 1.96, *SD* = 0.91). Parents reported moderately high use of the adaptive strategies Positive Reappraisal (*M* = 3.95, *SD* = 0.97) and Refocusing on Planning (*M* = 3.60, *SD* = 0.97), whereas the maladaptive strategy Catastrophizing was least endorsed (*M* = 2.89, *SD* = 1.21). Mean scores indicated moderate Positive Affect (*M* = 2.93, *SD* = 0.80) and relatively high Flourishing (*M* = 5.02, *SD* = 1.30). Perceived Parent-Child Closeness was high (*M* = 3.67, *SD* = 0.55), and Parent-Teacher Joining very high (*M* = 4.36, *SD* = 0.64).

**Table 1:** Descriptive statistics for main study variables (*N* = 212).

**Variable**	***M***	***SD***	**Skewness**	**Kurtosis**
COVID-19 perceived threat	3.29	0.97	−0.07	−0.54
Financial/material hardship	1.96	0.91	1.04	0.52
COVID-19 psychological impact	1.98	0.98	0.83	−0.09
Positive reappraisal	3.95	0.97	−0.94	0.68
Putting into perspective	2.96	1.12	0.12	−0.86
Refocusing on planning	3.60	0.97	−0.45	−0.21
Rumination	3.38	1.00	−0.43	−0.25
Catastrophizing	2.89	1.21	0.05	−0.94
Positive affect	2.93	0.80	0.10	−0.24
Flourishing	5.02	1.30	−0.80	0.03
Parent-child closeness	3.67	0.55	−1.59	2.46
Parent-teacher joining	4.36	0.64	−1.17	1.07

Means are calculated as the average of the scale items so that each score retains the original metric of its instrument. Range = 1–5, except Flourishing (1–7). Higher scores indicate higher levels of the construct.

**Table 2:** Zero-order Pearson correlations among main study variables and covariates (*N* = 212).

**Variable**	**1**	**2**	**3**	**4**	**5**	**6**	**7**	**8**	**9**	**10**	**11**	**12**	**13**	**14**	**15**	**16**
1. COVID-19 perceived threat	-															
2. Financial/material hardship	0.14^*^	-														
3. COVID-19 psychological impact	0.20^**^	0.38^***^	-													
4. Positive reappraisal	−0.06	−0.15^*^	−0.18^*^	-												
5. Putting into perspective	−0.19^**^	−0.09	−0.13	0.24^***^	-											
6. Refocusing on planning	−0.10	−0.20^**^	−0.22^**^	0.70^***^	0.26^***^	-										
7. Rumination	0.21^**^	0.02	0.16^*^	0.30^***^	−0.06	0.22^**^	-									
8. Catastrophizing	0.36^***^	0.14^*^	0.29^***^	0.10	−0.11	0.05	0.51^***^	-								
9. Positive affect	−0.28^***^	−0.25^***^	−0.36^***^	0.25^***^	0.25^***^	0.38^***^	−0.22^**^	−0.28^***^	-							
10. Flourishing	−0.18^**^	−0.28^***^	−0.36^***^	0.28^***^	0.20^**^	0.38^***^	−0.12	−0.25^***^	0.55^***^	-						
11. Parent-child closeness	−0.05	−0.33^***^	−0.29^***^	0.12	−0.05	0.18^**^	−0.07	−0.07	0.25^***^	0.42^***^	-					
12. Parent-teacher joining	−0.11	−0.31^***^	−0.23^***^	0.05	−0.09	0.05	0.04	−0.07	0.09	0.19^**^	0.48^***^	-				
13. Parent gender^*a*^	0.06	−0.04	0.03	−0.02	−0.10	−0.09	0.13	0.06	−0.09	−0.11	−0.04	0.10	-			
14. Parent age	0.01	−0.08	−0.10	0.08	0.12	0.03	0.06	−0.05	−0.00	0.08	0.05	0.06	−0.31^***^	-		
15. Socioeconomic status	−0.01	−0.47^***^	−0.19^**^	0.05	0.06	0.10	−0.11	−0.19^**^	0.25^***^	0.30^***^	0.21^**^	0.03	0.06	0.00	-	
16. Direct COVID-19 Experience^*b*^	0.06	0.01	0.09	0.05	0.05	0.09	0.12	0.18^**^	−0.06	−0.05	−0.10	−0.02	0.01	0.05	0.00	-

^a^0 = father, 1 = mother.

^b^0 = no, 1 = yes.

^*^p < 0.05.

^**^p < 0.01.

^***^p < 0.001.

Absolute skewness ranged from 0.05 to 1.59 and absolute kurtosis from 0.03 to 2.46, comfortably within the guidelines for robust maximum-likelihood estimation (|skew| < 2; |kurtosis| < 7; West et al., 1995). The largest departure was for Parent-Child Closeness (kurtosis = 2.46). A *z*-score inspection (|*z*| > 3.29) flagged five univariate outliers (2.4% of cases), and Mahalanobis-distance screening (*p* < 0.001) identified one multivariate outlier; removing these observations did not change any result, so all 212 cases were retained.

The three stressors were positively inter-correlated, the strongest being between Financial/Material Hardship and Psychological Impact, *r* = 0.38, *p* < 0.001; the association between Perceived Threat and Hardship was smaller yet significant, *r* = 0.14, *p* = 0.027. All stressors correlated positively with maladaptive strategies—except Hardship with Rumination, *r* = 0.02, *p* = 0.754. The largest effects were Perceived Threat with Catastrophizing, *r* = 0.36, *p* < 0.001, and Rumination, *r* = 0.21, *p* = 0.003, and Psychological Impact with Catastrophizing, *r* = 0.29, *p* < 0.001, and Rumination, *r* = 0.16, *p* = 0.018. Stressors showed small but significant negative correlations with selected adaptive strategies: Hardship with Positive Reappraisal, *r* = −0.15, *p* = 0.027, and Refocusing on Planning, *r* = −0.20, *p* = 0.003; Psychological Impact with Positive Reappraisal, *r* = − 0.18, *p* =0.011, and Refocusing on Planning, *r* = −0.22, *p* = 0.001; and Perceived Threat with Putting into Perspective, *r* = −0.19, *p* = 0.006. Adaptive strategies related positively to psychological wellbeing: Refocusing on Planning correlated moderately with Positive Affect and Flourishing (both *r*s = 0.38, *p*s < 0.001), and Positive Reappraisal and Putting into Perspective showed smaller yet significant links with both outcomes (|*r*|s = 0.20–0.28, *p*s ≤ 0.003). Maladaptive strategies were negatively related to wellbeing: Catastrophizing correlated with lower Positive Affect, *r* = −0.28, *p* < 0.001, and lower Flourishing, *r* = −0.25, *p* < 0.001; Rumination correlated modestly with lower Positive Affect, *r* = −0.22, *p* = 0.002.

In the relational domain, Financial/Material Hardship, *r* = −0.33, *p* < 0.001, and Psychological Impact, *r* = −0.29, *p* < 0.001, were negatively associated with Parent-Child Closeness and likewise with Parent-Teacher Joining (Hardship *r* = −0.31, Impact *r* = −0.23, *p*s ≤ 0.001). Among the five regulation strategies, only Refocusing on Planning correlated with a relational outcome, showing a small positive link with Parent-Child Closeness, *r* = 0.18, *p* = 0.009. The two relational indicators were strongly correlated, *r* = 0.48, *p* < 0.001. Socioeconomic Status correlated negatively with Financial/Material Hardship, *r* = −0.47, *p* < 0.001, and positively with Positive Affect, Flourishing, and Parent-Child Closeness (|*r*|s = 0.21–0.30, *p*s ≤ 0.002). Parent gender correlated negatively with age, *r* = −0.31, *p* < 0.001, but showed no significant associations with the focal psychological or relational variables; likewise, age and direct COVID-19 experience did not correlate significantly with the central constructs. Taken together, these correlations depict a coherent pattern whereby greater pandemic-related stress is linked to heavier reliance on maladaptive emotion-regulation strategies and with poorer psychological and relational functioning, supporting the decision to test all five cognitive emotion-regulation strategies as mediators in subsequent structural models.

### 3.2 Structural mediation analysis

Adhering to the four-step procedure described in the Data Analytic Plan, we first estimated a fully specified model (M1) that included every direct path from the three pandemic stressors to the two latent outcomes and every indirect path through the five emotion-regulation strategies. We then imposed progressively stricter constraints. Constraining all direct stressor → outcome paths to zero (M2) worsened fit markedly. Modification indices pointed to two necessary direct paths (Psychological Impact → Psychological Wellbeing and Financial/Material Hardship → Relational Functioning). Refreeing these paths produced a partial-mediation model (M3) whose fit did not differ statistically from M1. Finally, all remaining non-significant paths were fixed to zero, yielding a parsimonious solution (M4) that retained excellent fit and did not differ significantly from M1 (see Table 3).

**Table 3:** SEM model fit and comparisons (M1–M4).

**Model**	**χ^2^ (*df*)**	***P***	**CFI**	**RMSEA**	**SRMR**	***Δχ*^2^ (*df*) vs. M1**	***P*Δ**	**ΔCFI vs. M1**	**ΔRMSEA vs. M1**	**ΔSRMR vs. M1**
M1	39.90 (25)	0.030	0.972	0.053	0.025	—	—	—	—	—
M2	66.95 (31)	< 0.001	0.933	0.074	0.047	22.76 (6)	< 0.001	−0.039	+0.021	+0.022
M3	47.61 (29)	0.016	0.965	0.055	0.030	7.39 (4)	0.117	−0.007	+0.002	+0.005
M4	93.69 (84)	0.220	0.982	0.023	0.058	55.04 (59)	0.622	+0.010	−0.030	+0.033

**Figure 2:**
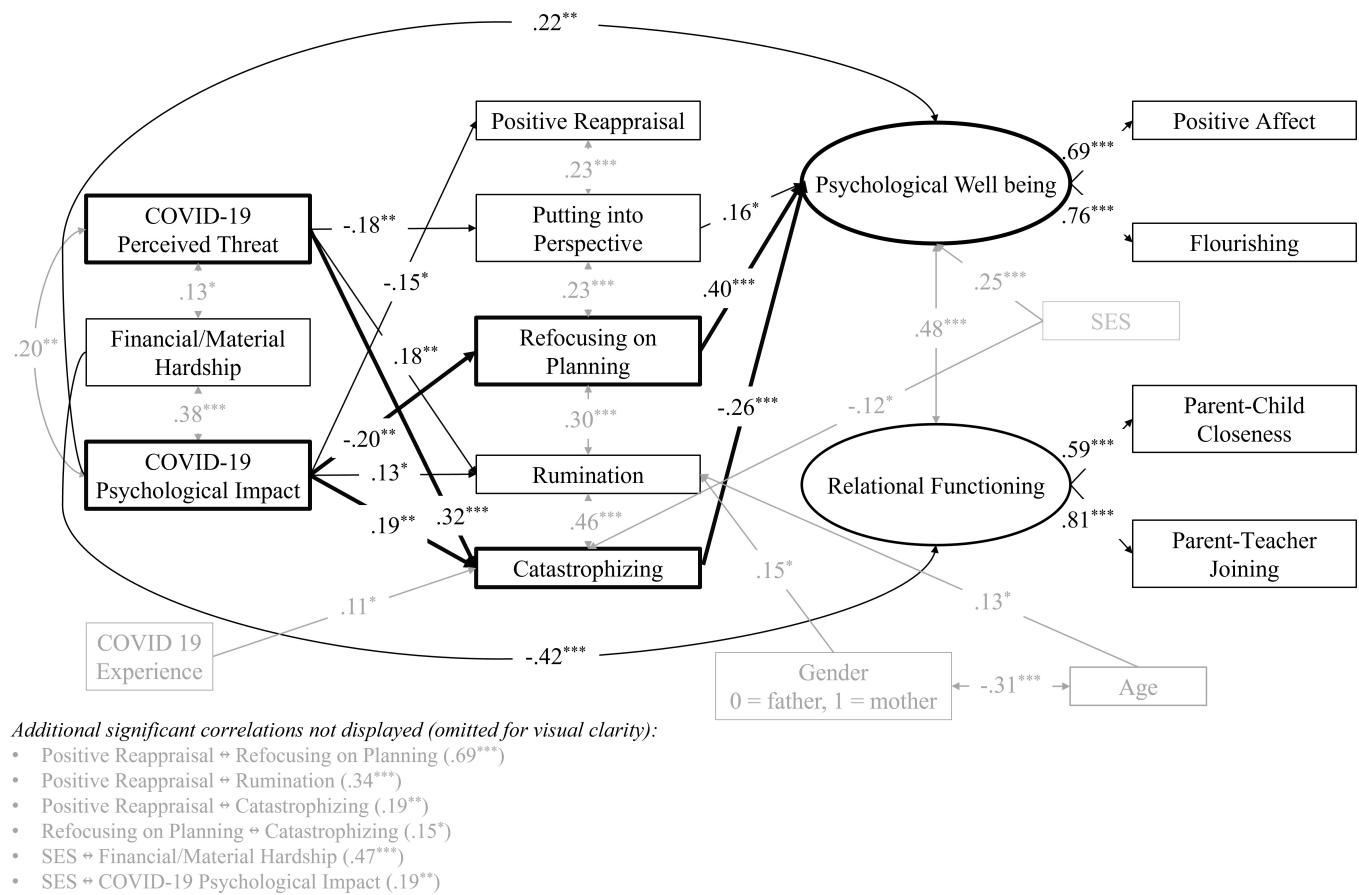
Final estimated model. Black arrows represent focal study variables and their estimated paths; gray arrows denote control variables, their paths, and inter-correlations. Paths and variable labels involved in statistically significant indirect effects are shown in bold. Standardized regression coefficients (β) are reported; residual variances are omitted for clarity. SES, socio-economic status. ^*^p < 0.05, ^**^p < 0.01, ^***^p < 0.001.

Figure 2 displays the standardized estimates for the final model. Among the three stressors, Financial/Material Hardship retained an exclusive direct link with lower Relational Functioning (β = −0.42, *p* < 0.001). Psychological Impact was associated with Psychological Wellbeing both directly (β = −0.22, *p* = 0.010) and indirectly through its relations with emotion-regulation strategies (see below). COVID-19 Perceived Threat did not show a direct association with either latent outcome, but it exhibited one of the strongest indirect patterns via cognitive emotion regulation.

Regarding the mediators, three strategies emerged as central. Maladaptive Catastrophizing was positively predicted by Perceived Threat (β = 0.32, *p* < 0.001) and Psychological Impact (β = 0.19, *p* = 0.003) and, in turn, was negatively related to Psychological Wellbeing (β = −0.26, *p* < 0.001). Rumination followed a similar, albeit weaker, pattern (Threat → Rumination, β = 0.18, *p* = 0.009; Impact → Rumination, β = 0.13, *p* = 0.039), but its downstream path to Wellbeing was trimmed for non-significance. On the adaptive side, Refocusing on Planning was negatively associated with Psychological Impact (β = −0.20, *p* = 0.001) and positively associated with Wellbeing (β = 0.40, *p* < 0.001). Putting into Perspective was also negatively linked to Perceived Threat (β = −0.18, *p* = 0.010) and showed a modest positive relation with Wellbeing (β = 0.16, *p* = 0.021). Positive Reappraisal, although negatively predicted by Psychological Impact (β = −0.15, *p* = 0.030), was not associated with either outcome.

Tests of indirect associations supported these patterns. Perceived Threat showed a significant total indirect association with lower Psychological Wellbeing (β = −0.11, *p* = 0.001), driven primarily by the Catastrophizing pathway (β = −0.08, *p* = 0.005); the specific route through Putting-into-Perspective alone was not significant (*p* = 0.095). Psychological Impact displayed a comparable total indirect association with lower Wellbeing (β = −0.13, *p* < 0.001), operating through Catastrophizing and Refocusing on Planning in addition to its direct link. After non-significant parameters were trimmed, no indirect association linked Financial/Material Hardship to either latent outcome.

Among the covariates, Socioeconomic Status remained a positive predictor of Psychological Wellbeing (β = 0.25, *p* < 0.001). Parent age, gender, and direct COVID-19 experience were unrelated to the latent outcomes once the primary pathways were accounted for.

In combination, the final model explained 49.6% of the variance in Psychological Wellbeing and 18.0% in Relational Functioning. The pattern suggests that the cognitive interpretation parents assigned to the pandemic, expressed in catastrophizing thoughts and reduced plan-oriented coping, linked perceived threat and psychological impact to their wellbeing, whereas the more tangible strain of financial hardship bypassed these cognitive processes and directly associated with family-school relationships.

## 4 Discussion

The present study aimed to examine the mediating role of cognitive emotion regulation strategies in the associations between COVID-19-related stressors (i.e., perceived threat, psychological distress, and financial hardship) and parents' psychological and relational adjustment, the latter including both parent-child and parent-teacher relationships. In line with the hypotheses, the results revealed nuanced patterns of direct and indirect associations. Specifically, perceived threat related to COVID-19 and pandemic-connected psychological distress were indirectly associated with parents' psychological wellbeing through emotion regulation strategies, highlighting these cognitive processes as potential means linking stress to psychological adjustment outcomes. Among the cognitive strategies considered, catastrophizing, defined as persistent negative thoughts on worst-case scenarios, emerged as a salient maladaptive mediator. Parents who reported greater COVID-19 -related threat and psychological distress also reported engaging in higher levels of catastrophizing, which in turn was associated with lower psychological wellbeing. Conversely, the adaptive strategy of refocusing on planning, involving structured and goal-oriented coping efforts, mediated the association between COVID-19-related psychological distress and better psychological outcomes. Parents who reported higher use of this pragmatic coping strategy tended to report better psychological functioning, suggesting its potentially beneficial role during pandemic-induced stress. By contrast, financial hardship, reflecting tangible economic challenges experienced during the pandemic, showed no significant indirect associations through the emotion regulation strategies assessed. Instead, this stressor exhibited direct negative associations with relational adjustment, particularly in parent-child closeness and parent-teacher relationship quality. Greater financial hardship was directly related to lower perceived relationship quality, independent of the cognitive coping processes considered. The mediation model accounted for approximately half of the variance in psychological adjustment, whereas a more modest proportion (about one-fifth) of the variance was explained for relational adjustment outcomes. Collectively, these results highlight the relevance of cognitive emotion regulation as a mediator in psychological adjustment but suggest a limited role of these strategies regarding relational outcomes in contexts characterized by severe economic stressors. Thus, our findings provide further insights into family resilience dynamics, emphasizing that cognitive coping may have limits in mediating relational outcomes against significant external stressors such as economic hardship.

These findings align closely with several established theoretical frameworks addressing stress, coping, and family functioning. Firstly, the Family Stress Model (Masarik and Conger, 2017) provides a pertinent conceptual framework for interpreting these results. This approach posits that external stressors, such as economic hardship or health-related concerns, negatively influence family outcomes primarily through their impact on caregivers' emotional states and coping processes. In accordance with this perspective, our findings indicate that perceived COVID-19 threat and psychological distress were indirectly associated with parental psychological outcomes via cognitive emotion regulation strategies, specifically catastrophizing and refocusing on planning. These results are consistent with Family Stress Model's emphasis on internal cognitive and emotional processes as critical mediators linking external stressors to family adjustment. Additionally, the direct association we observed between financial hardship and relational outcomes resonates strongly with the recognition that certain tangible stressors, especially economic pressures, may directly relate to family functioning, irrespective of cognitive coping strategies (Negi and Sattler, 2025). Thus, our findings extend the relevance of the Family Stress Model to the context of the COVID-19 pandemic, providing empirical support for the idea that external stressors may differentially relate to psychological vs. relational outcomes.

Furthermore, the transactional model of stress and coping (Lazarus and Folkman, 1984) offers additional interpretive clarity regarding the current results. This model underscores the importance of cognitive appraisals and coping responses as potential mediators in the stress-adjustment association. Our results indicate that maladaptive appraisal processes, particularly catastrophizing, were associated with poorer psychological outcomes, while more adaptive, proactive coping responses, such as refocusing on planning, were associated with better psychological functioning. Thus, these findings are consistent with transactional perspectives suggesting that individuals' subjective interpretations and responses to stressors may significantly mediate their adjustment outcomes. However, in line with transactional thinking, it remains important to acknowledge the bidirectional and dynamic nature of these associations over time, which our cross-sectional data cannot directly assess (Sameroff, 2009).

The concept of regulatory flexibility (Bonanno and Burton, 2013) further complements these findings. Regulatory flexibility proposes that adaptive responses to stress depend on an individual's ability to select and apply emotion regulation strategies effectively according to contextual demands. Our findings suggest that parents reporting more frequent use of structured, pragmatic strategies like planning also reported better psychological adjustment, whereas those reporting higher levels of rigid and maladaptive coping responses, such as catastrophizing, reported poorer outcomes. These observations are in line with the regulatory flexibility framework, indicating that flexible deployment of coping strategies relevant to the context might play a significant mediating role in psychological adjustment during extended stressors such as the COVID-19 pandemic.

Finally, integrative multi-systemic resilience models (Liu et al., 2017; Walsh, 2020) offer valuable insight into the complex interplay between individual coping capacities and external contextual influences. Our findings suggest that cognitive emotion regulation strategies represent individual-level processes that may mediate psychological outcomes during stress. At the same time, the observed direct associations between financial hardship and relational outcomes highlight that external contextual factors may also be critical to family resilience, potentially influencing relational adjustment independently from internal coping processes. These findings align well with multi-systemic resilience frameworks, which advocate comprehensive approaches that simultaneously address internal psychological resources and external environmental supports to facilitate family adaptation in contexts characterized by high stress and adversity.

Beyond these theoretical frameworks, the present findings also echo into a growing body of empirical evidence that has documented similar patterns in families exposed to prolonged stress, both prior to and during the COVID-19 pandemic. Previous research has consistently identified cognitive emotion regulation strategies as mediators between stress exposure and psychological adjustment (Aldao et al., 2010; Manesh and Malak, 2025; Martin and Dahlen, 2005). Consistent with this extensive literature, the present study found significant indirect associations linking COVID-19-related stressors to parental psychological outcomes via emotion regulation strategies. Specifically, as previously mentioned, catastrophizing emerged as a key maladaptive mediator associated with poorer psychological wellbeing. This result is consistent with findings from Prikhidko et al. (2020) and Wang et al. (2021), who reported strong links between maladaptive cognitive strategies (such as catastrophizing and rumination) and heightened psychological suffering during the COVID-19 crisis. Similarly, our findings, highlighting the adaptive role of structured problem-focused coping strategies, such as planning, align closely with prior evidence demonstrating beneficial associations between structured coping and psychological adjustment in prolonged stressful contexts (Restubog et al., 2020). This congruence suggests that pragmatic, solution-oriented coping may be particularly relevant for managing psychological stress associated with crises characterized by high uncertainty and practical challenges, such as COVID-19 lockdowns and restrictions. However, our observation that positive reappraisal and rumination did not significantly mediate the associations between stressors and psychological outcomes diverges somewhat from prior research findings (e.g., Xu et al., 2020). This absence of indirect associations for positive reappraisal and rumination is compatible with several considerations. First, in parallel-mediator models, correlated strategies can compete statistically; strategies explaining more variance (here, planning and catastrophizing) may attenuate the unique indirect contribution of others when estimated simultaneously (Aldao et al., 2010; Garnefski and Kraaij, 2006). Second, contextual fit likely matters: during prolonged, partly uncontrollable disruptions, action-oriented coping (planning) and lower catastrophizing may relate more proximally to positive wellbeing once competing pathways are considered; this interpretation aligns with regulatory-flexibility accounts and COVID-19 coping evidence (Bonanno and Burton, 2013; Bonanno et al., 2024; Fluharty and Fancourt, 2021; Haver et al., 2023). Third, our outcomes emphasized hedonic/eudaimonic wellbeing rather than symptoms; rumination tends to show stronger links with negative affect/internalizing phenotypes, which can reduce its incremental association with flourishing or positive affect when modeled alongside catastrophizing (Aldao et al., 2010; see also Xu et al., 2020). Fourth, measurement may play a role: brief CERQ subscales capture typical tendencies but can have limited sensitivity to detect small unique indirect effects under multivariate competition (Garnefski and Kraaij, 2006; Hasani et al., 2024). Finally, population/context specificity is plausible: evidence from distinct adult groups (e.g., retirees; incarcerated adults) suggests that the salience of particular strategies varies with role demands, setting, and stressor profiles (Khartoomi and Khedmatgozar, 2024; Manesh and Malak, 2025).

Regarding relational adjustment, our findings converge with existing research underscoring the associations between financial hardship and relational outcomes (Conger et al., 1992). Indeed, economic strain has been repeatedly linked with decreased relational quality and increased family conflict across various stress contexts (Conger and Conger, 2002). Our findings align with these prior studies by identifying direct associations between pandemic-related financial hardship and lower parent-child closeness and diminished parent-teacher relationship quality, suggesting that economic stress may have implications for relational dynamics irrespective of the cognitive emotion regulation strategies employed by parents and supporting distinctive pathways for the relational and individual outcomes. Nevertheless, whether economic stress affects, or not, individual functioning remains an open question, as measurement issue might be part of the explanation of these results: Chan et al. (2024) systematic review on the relation between socioeconomic strain during the COVID-19 pandemic and psychological health showed that only half of studies confirmed such relation. Given the variety of socio-economic indicators used, the authors highlight the complexity of operationalizing and measuring such a multidimensional construct. In our work, we considered a single construct of financial distress, which might explain the absence of effects on an individual's psychological functioning.

Additionally, while limited, existing research on family-school interactions during COVID-19 underscores increased relational strain between parents and teachers due to pandemic-related disruptions (Carrión Martínez et al., 2021). Our study adds further detail to this literature, suggesting that economic stressors might uniquely relate to reduced parent-teacher collaboration. Thus, our results support recent suggestions (Keengwe and Onchwari, 2024; Graham et al., 2021; Sengönül, 2022). that family-school partnerships may be particularly vulnerable to disruption under economic stress, irrespective of parental coping strategies.

Collectively, the current findings reinforce and extend prior evidence on family coping during crises by confirming the critical mediating role of cognitive emotion regulation strategies for parental psychological adjustment: while existing findings show that emotion regulation mediates the impact of COVID-19-related stressors on a variety of individual indicators of mental health (e.g., Dai et al., 2021; García-Batista et al., 2021; Ursu and Măirean, 2022), the presents study adds the novelty of testing such indirect relations also in the prediction of relational functioning. Our findings add nuance by highlighting that these relational outcomes may not be substantially mediated by these cognitive strategies, particularly when families face significant external stressors such as economic hardship. Thus, the present study contributes to a growing literature seeking to clarify the specific conditions and contexts under which emotion regulation strategies may or may not mediate associations between stress and various dimensions of family functioning.

### 4.1 Practical implications for intervention and policy

Our findings offer a number of implications for interventions and policies aimed at supporting parents and families who experience significant stressors. These implications are particularly relevant for clinical practice, educational settings, and public policy. Within a clinical framework, the evidence from this study suggests that emotion regulation strategies represent important processes that can be addressed in parent-focused interventions. Because catastrophizing emerged as a key maladaptive strategy, clinical approaches that explicitly address this cognitive style may be useful. Indeed, evidence shows that dysfunctional emotion regulation strategies can be improved with brief clinical interventions (i.e., Khartoomi and Khedmatgozar, 2024). Cognitive-behavioral interventions, for instance, can guide parents to identify and modify catastrophic patterns of thought, replacing them with more balanced interpretations and, when appropriate, strategies focused on concrete action planning. Additionally, the significant indirect associations involving planning suggest that clinicians might promote structured and goal-oriented coping among parents as a way to strengthen their psychological wellbeing during stressful conditions. Such strategies could include developing stepwise action plans, identifying practical resources, and setting attainable goals. Interventions aimed at fostering flexibility in selecting coping strategies (helping parents to shift away from rigid negative patterns and toward more adaptive ones) are particularly aligned with the evidence reported here and with frameworks that emphasize flexible coping (Bonanno and Burton, 2013; Bonanno et al., 2024).

The findings also underline the importance of attention to parents' psychological wellbeing in school contexts. School-based professionals, such as teachers, school psychologists, and counselors, can play a valuable role by offering parents information and guidance regarding stress and coping during times of disruption. When families experience significant stress, their ability to collaborate with teachers and remain engaged in their children's education may be compromised. Schools can respond proactively by creating structured opportunities for communication with parents, providing clear guidance for learning tasks, and ensuring that resources are available for families that require additional support. Moreover, educators can be trained in approaches that recognize signs of parental strain, using a supportive and empathetic stance to maintain constructive parent-teacher relationships even under challenging circumstances. With this respect, Teacher Training Programs (TTP) focused on family engagement may be useful tools to pursue this aim: family engagement has to do with how teachers support parents in implementing effective school-related activities at home (i.e., supporting literacy at home, monitoring homework completion) and embrace approaches used collaboratively to promote partnerships between families and schools (i.e., family–school partnerships). Meta-analytic evidence shows that Family Engagement TTPs have positive effects on teachers' attitudes, knowledge, and practices related to family engagement, particularly when the intervention is focused on collaborative planning and problem solving, communication strategies, cultural awareness, family-engagement attitudes and beliefs, and parent–teacher relationships (Smith and Sheridan, 2019). Beyond the individual and school levels, the results carry implications for policy and community-level interventions. The direct associations between financial hardship and relational outcomes indicate that addressing economic strain represents an essential component of supporting family systems during large-scale stress events. Policies that provide timely financial support, access to social services, and resources for parents may reduce the relational difficulties that are frequently associated with economic hardship. Importantly, such interventions should not be limited to emergency periods but integrated into broader social systems to ensure that vulnerable families receive sustained support. Finally, community mental health initiatives (such as outreach programs, workshops, or digital resources) can contribute to the dissemination of knowledge about effective coping strategies and facilitate access to psychological support for parents.

These implications suggest that interventions at multiple levels—psychological, educational, and structural—are likely to be most effective when they are integrated. The combined promotion of adaptive coping skills and provision of external resources can support family resilience in contexts characterized by both chronic and acute stressors.

### 4.2 Limitations and future directions

While the present study adds to the understanding of how COVID-19-related stressors are associated with parents' psychological and relational adjustment, several limitations need to be acknowledged. These limitations also point toward directions for future research. A first limitation concerns the cross-sectional design of the study. The analyses were based on data collected at a single time point, which restricts the ability to draw conclusions about the ways of causation and examine how associations between stressors, emotion regulation strategies, and outcomes develop and potentially change over time. Longitudinal studies are, therefore, needed to better explore the temporal ordering of these associations and to clarify whether the patterns found here remain stable, diminish, or intensify as stressors evolve. A longitudinal approach would also allow the investigation of reciprocal processes, for instance whether parents' difficulties in regulating emotions might be associated with later increases in stress, or conversely whether prolonged exposure to stress could influence subsequent coping strategies. Also, longitudinal approaches would allow to understand possible long-term effects of each emotion regulation strategy: catastrophizing is a well-recognized transdiagnostic process involved in the etiopathogenesis of many mental disorders (Gellatly and Beck, 2016); on the other side, refocusing on planning predicts the reduction, from before the pandemic to during the 2020 lockdown, of the perceived emotional burden (Sacchi and Dan-Glauser, 2021). Therefore, it is plausible to suggest that each of these emotion regulation strategies might lead to dysfunctional and functional outcomes over time.

Second, self-report measures were used for all variables. Although validated instruments were employed, reliance on single-informant data introduces the possibility of shared method variance and may not fully capture the complexity of the constructs examined. Future research would benefit from the inclusion of multiple informants, such as children, teachers, and partners, as well as complementary data sources, such as behavioral observations and administrative records (for example, frequency of contact with teachers). A multi-method multi-informant approach would strengthen the validity of findings and provide a more complete picture of family functioning.

Third, the sample characteristics impose limitations on generalizability. Participants were predominantly mothers from Southern Italy, where mothers have been shown to be primary attachment figures for their children (Cassibba et al., 2017). This setting is characterized by strong familistic orientations and dense kin networks that can buffer stress, but also by traditional role expectations that concentrate caregiving and school-related tasks within families, with gendered divisions of care intensified during the pandemic (Bacchini et al., 2024; Bosoni, 2024; Del Boca et al., 2020). During COVID-19, Italian families faced prolonged school closures and hybrid arrangements, with intensive parent-mediated learning demands and variable parent–teacher coordination (Benigno et al., 2020; Gandini et al., 2021; OECD, 2020; Trentin et al., 2020). These cultural and institutional features are consistent with the pattern observed: financial strain showed stronger associations with relational outcomes, whereas cognitive strategies showed stronger associations with psychological wellbeing. In familistic contexts, economic stressors may more immediately burden day-to-day relationships with children and schools, whereas individual coping may be more closely linked to subjective wellbeing. At the same time, the predominance of mothers suggests that the observed relations may reflect maternal experiences more than paternal or other caregiver profiles. Although parent gender was included as a covariate, the compositional imbalance and the region-focused community recruitment limit population-level inference. Accordingly, within this context, the present associations should be interpreted as context-embedded: they are likely conceptually general, yet the magnitude and salience of specific paths may vary across countries with different welfare regimes, school–family interfaces, and cultural norms regarding caregiving and familism, and may also vary by caregiver gender and division of caregiving responsibilities. Future studies should recruit more diverse caregiver samples, including balanced mother-father subsamples and other caregivers, and pursue cross-national replications to assess generalizability across cultural and socio-economic contexts.

Another limitation concerns the focus on cognitive strategies as measured by the instrument employed. Emotion regulation includes a wide range of behavioral, interpersonal, and physiological strategies that were not included in the present study. Future research would benefit from considering these additional dimensions, such as expressive suppression, seeking social support, or physiological regulation, to provide a more comprehensive view of parental coping. Moreover, the study did not include measures of regulatory flexibility (as noted earlier, the ability to adapt strategies to changing circumstances) which might be a particularly relevant construct in periods characterized by rapid and unpredictable stressors.

Finally, although the current study considered psychological wellbeing and relational adjustment as outcomes, other important domains remain to be investigated. Future research could extend the model to include parenting behaviors, children's wellbeing, and indicators of academic and social functioning. Examining these broader outcomes would be particularly relevant for understanding the implications of parents' coping processes for family systems as a whole. Future studies could also test intervention models directly derived from these findings. For example, interventions designed to reduce maladaptive cognitive strategies and promote planning and other structured coping approaches could be evaluated in terms of their effects on psychological and relational outcomes. Such studies would not only confirm the theoretical importance of these strategies but also provide practical insights for designing effective family support programs during periods of crisis.

## 5 Conclusions

In summary, the present study offers new insights into how parents' cognitive emotion regulation strategies are related to their adjustment in the context of significant stressors such as those experienced during the COVID-19 pandemic. The mediation model tested in this study indicated that maladaptive strategies, particularly catastrophizing, were indirectly associated with poorer psychological wellbeing, whereas the use of adaptive, structured strategies such as planning showed indirect associations with more favorable psychological outcomes. At the same time, financial hardship demonstrated direct associations with relational adjustment, pointing to the potential limits of these individual-level strategies in the face of concrete and severe external stressors.

Interpreted through established theoretical frameworks (including the Family Stress Model, transactional models of stress and coping, regulatory flexibility, and multi-system resilience perspectives) these findings contribute to a more nuanced understanding of the processes through which stress and coping are related to parental functioning. The results are broadly consistent with previous research, while adding specificity by identifying the mediating role of certain cognitive strategies and by highlighting domains (such as relational outcomes) where these strategies appear less central.

Overall, these findings support the importance of considering both individual cognitive resources and contextual factors when addressing family adaptation in times of widespread stress. By promoting adaptive coping skills while also addressing material stressors through supportive clinical, educational and policy measures, professionals and decision-makers can strengthen the psychological wellbeing of parents and foster more constructive family and school relationships. Such integrated, multi-level approaches are essential for enhancing family resilience and preparedness for future collective challenges.

## Data Availability

The datasets presented in this study can be found in online repositories. The names of the repository/repositories and accession number(s) can be found at: https://figshare.com/s/342bbcfd9c5986868331.
